# Colorectal surgery simulation in realistic models: experience of Brazilian residents

**DOI:** 10.1590/0102-672020260000039e1968

**Published:** 2026-09-28

**Authors:** Carolina Reis BONIZZIO, Carlos Frederico Sparapan MARQUES, Helio MOREIRA, Luiz Fernando Ferraz da SILVA, Ignacio OTOCH, Paulo HERMAN, Ulysses RIBEIRO

**Affiliations:** 1 Universidade de São Paulo, Faculty of Medicine, Department of Gastroenterology, Digestive System Surgery, Coloproctology Division – São Paulo (SP), Brazil.; 2 Universidade Federal de Goiás, Faculty of Medicine, Coloproctology Service – Goiânia (GO), Brazil.; 3 Universidade de São Paulo, Faculty of Medicine, Department of Pathology – São Paulo (SP), Brazil.; 4 Universidade de São Paulo, Faculty of Medicine, Department of Surgery – São Paulo (SP), Brazil.

**Keywords:** Simulation training, Colorectal surgery, Learning, Internship and residency, Treinamento por simulação, Cirurgia colorretal, Aprendizagem, Internato e residência

## Abstract

**Background:**

Practical training is essential in surgical education, and simulation is an effective strategy for developing technical skills in a safe environment. This study describes the experience of the Advanced Practical Course in Colorectal Surgery, which enrolled 52 colorectal surgery residents from different regions of Brazil. The course provided supervised training in colorectal procedures using realistic models, including synthetic simulators, animal models, and fresh cadavers.

**Aims:**

To evaluate the applicability of a theoretical-practical training method for first-year colorectal surgery residents in Brazil, as well as theoretical knowledge acquisition, self-confidence in performing procedures, and participant satisfaction.

**Methods:**

This study describes the experience of the Advanced Practical Course in Colorectal Surgery, which enrolled 52 colorectal surgery residents from different regions of Brazil. The course provided supervised training in colorectal procedures using realistic models, including synthetic simulators, animal models, and fresh cadavers.

**Results:**

The course was divided into two phases: (1) theoretical lectures and intraoperative videos delivered through a live online platform; and (2) two days of surgical simulation using cadaveric, porcine, and synthetic models under the supervision of experienced colorectal surgeons. Objective theoretical assessments were performed before and after each phase, along with subjective evaluations at the beginning and end of the course. Statistical analysis was conducted using R Commander^®^ software. A significant improvement in self-confidence was observed for laparoscopic right colectomy (p=0.0043), laparoscopic left colectomy (p=0.00048), and total mesorectal excision (p=0.00023). Technical knowledge increased after both the theoretical (p=0.0113) and practical (p=0.00023) phases. Overall satisfaction was high, with a mean score of 9.3 out of 10.

**Conclusions:**

The theoretical-practical simulation-based method proved to be applicable and effective, leading to improved knowledge acquisition, increased procedural confidence, and high satisfaction among first-year colorectal surgery residents.

## ARTICLE HIGHLIGHTS

Hybrid simulation training improved knowledge and confidence in colorectal surgery residents.

Realistic models (cadaveric, porcine, synthetic) enabled safe surgical skill development.

Confidence significantly increased in key laparoscopic procedures (colectomy and TME).

High satisfaction (9.3/10) supports simulation as an effective training strategy.

## Summary

**CENTRAL MESSAGE:**

This study shows how hands-on training can improve surgical education. We evaluated an advanced practical course for first-year colorectal surgery residents in Brazil, combining online classes with realistic surgical simulation using synthetic models, animals, and fresh cadavers. After the course, residents showed better theoretical knowledge, greater confidence to perform procedures, and high satisfaction. Our experience suggests that structured simulation-based training is effective and accessible way to prepare young surgeons for colorectal surgery.

**PERSPECTIVES:**

The Advanced Practical Course in Colorectal Surgery demonstrated that innovative, hands-on education can significantly improve the training of colorectal surgery residents in Brazil. By combining realistic simulation models with a hybrid teaching approach that integrates theory and practice, the course allowed residents to safely develop essential technical skills and build confidence. This experience highlights the value of structured, high-quality surgical training outside the operating room. Expanding and maintaining similar programs is crucial to broaden access to advanced education and to support the development of new centers of excellence in surgical training throughout the country.

## VISUAL ABSTRACT

**Figure:**
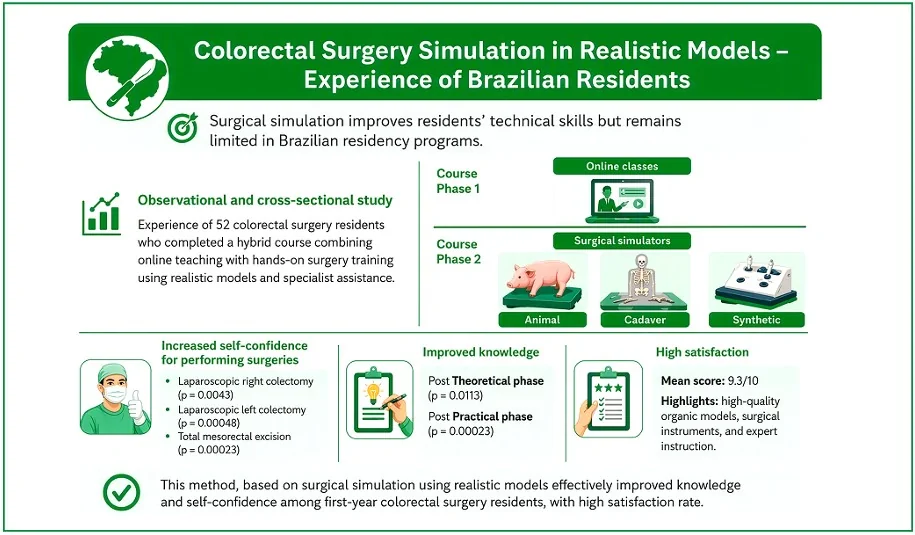

## RESUMO

**Racional:**

O treinamento prático é essencial na formação cirúrgica, e a simulação é uma estratégia eficaz para o desenvolvimento de habilidades técnicas em um ambiente seguro. Este estudo descreve a experiência do Curso Prático Avançado em Cirurgia Colorretal, que contou com a participação de 52 residentes de cirurgia colorretal de diferentes regiões do Brasil. O curso ofereceu treinamento supervisionado em procedimentos colorretais utilizando modelos realistas, incluindo simuladores sintéticos, modelos animais e cadáveres frescos.

**Objetivos:**

Avaliar a aplicabilidade de um método de treinamento teórico-prático para residentes do primeiro ano de Coloproctologia no Brasil, bem como a aquisição de conhecimento teórico, a autoconfiança na realização de procedimentos, e a satisfação dos participantes.

**Resultados:**

O curso foi dividido em duas fases: (1) aulas teóricas e vídeos intraoperatórios ministrados por meio de uma plataforma online ao vivo; e (2) dois dias de simulação cirúrgica utilizando modelos cadavéricos, suínos e sintéticos sob a supervisão de cirurgiões colorretais experientes. Avaliações teóricas objetivas foram realizadas antes e depois de cada fase, juntamente com avaliações subjetivas no início e no final do curso. A análise estatística foi conduzida utilizando o software R Commander ^®^. Observou-se uma melhora significativa na autoconfiança para colectomia direita laparoscópica (p=0,0043), colectomia esquerda laparoscópica (p=0,00048) e excisão total do mesorreto (p=0,00023). O conhecimento técnico aumentou após as fases teórica (p=0,0113) e prática (p=0,00023). A satisfação geral foi elevada, com uma pontuação média de 9,3 em 10.

**Conclusões:**

O método teórico-prático baseado em simulação mostrou-se aplicável e eficaz, levando a uma melhor aquisição de conhecimento, aumento da confiança nos procedimentos e alta satisfação entre os residentes de Coloproctologia do primeiro ano.

## INTRODUCTION

 The training of surgeons during residency requires exposure to situations that enable not only critical evaluation in the pre-, intra-, and postoperative care, but also mastery of operative technique^21^ . Among the essential competencies are anatomical knowledge, the execution of surgical steps, and the ability to handle instruments, especially in minimally invasive modalities. The use of simulators allows practice in a controlled and safe environment before operating on patients20,21, promoting progressive learning and confidence of a resident under supervision^2,10,20^ . 

 The Brazilian Society of Coloproctology (BSC) and the Faculty of Medicine of the University of São Paulo (FMUSP), which are committed to providing access to high-quality education, offered an unprecedented nationwide course for all colorectal surgery residents^3^ . Predominantly practical, the course — organized by the divisions of Digestive System Surgery, Coloproctology, Urology, Pathology, and Surgical Technique — provided training in colorectal procedures performed on synthetic and organic models (animal and cadaver) under the guidance of BSC-certified specialists. 

 This study describes Brazil’s pioneering experience in teaching colorectal surgery to first-year residents using contemporary surgical education methodologies, with an emphasis on practical activities using wet models, which remain limited in most colorectal surgery residency programs. 

 The primary objective of this study was to evaluate the applicability of a novel university-based, theoretical-practical model for teaching colorectal surgery to first-year colorectal surgery residents in Brazil. 

 Secondary objectives were to evaluate theoretical knowledge acquisition, the evolution of confidence in performing procedures, and participant satisfaction after the course, measured using a specific and dedicated questionnaire. 

## METHODS

 As part of the continuing education program of the Brazilian Society of Coloproctology, the Advanced Practical Course in Colorectal Surgery was held on June 24–25, 2024, in São Paulo, and was offered to first-year colorectal surgery residents from all regions of Brazil. A total of 52 residents participated. The pedagogical model adopted was hybrid, divided into two phases: Phase 1 “Pre-Course”— theoretical classes and presentation of intraoperative videos in a live online platform. Phase 2 “In-Person Course”— two days of surgical simulation using cadaveric, porcine, and synthetic models under the supervision of colorectal surgeons. The model is detailed below: 

### Phase 1 – “Pre-Course”

 Two weeks before the in-person event, residents participated in a live online theoretical module entitled Pre-Course. Seven classes of approximately 15 minutes each were delivered, covering anatomical review, technical tips, and intraoperative videos illustrating laparoscopic procedures including right and left colectomy, rectosigmoidectomy with total mesorectal excision, and intra- and extracorporeal anastomoses. Between classes, faculty answered questions and stimulated interaction through guided prompts. 

 Additionally, for study purposes, edited videos demonstrating standardized surgical steps on cadavers — produced by medical students and members of the Faculty of Medicine of Universidade de Sao Paulo (FMUSP) Coloproctology League under supervision — were provided. These materials, later presented at academic conferences (4), served as supplementary study aids. 

### Phase 2 – “In-Person Course”

 The in-person course occurred across two locations: FMUSP and Medtronic Brasil ^®^, with rotating groups. 

 FMUSP: Teams of three to four residents accompanied by one instructor performed laparoscopic right and left colectomies on fresh cadaveric models and mesorectal dissection with mechanical anastomosis on porcine models. Each practical session lasted approximately four hours. Across two days, four sessions were conducted (Figure 1), using a total of 16 fresh cadavers (provided by São Paulo Autopsy Service of the Universidade de São Paulo (SVOC-USP), with family authorization). At the end of the activities, the cadavers were transferred, as routine, for pathologic evaluation after record of findings and procedures performed. A total of 16 pigs were used; all animals were anesthetized by a licensed veterinarian, and subsequently euthanized in accordance with ethical regulations. There was a preference for selecting male adult animals to facilitate pelvic dissection due to the absence of the uterus and adnexa. During practical sessions, previously provided procedural videos were continuously displayed for consultation. 

**Figure 1:**
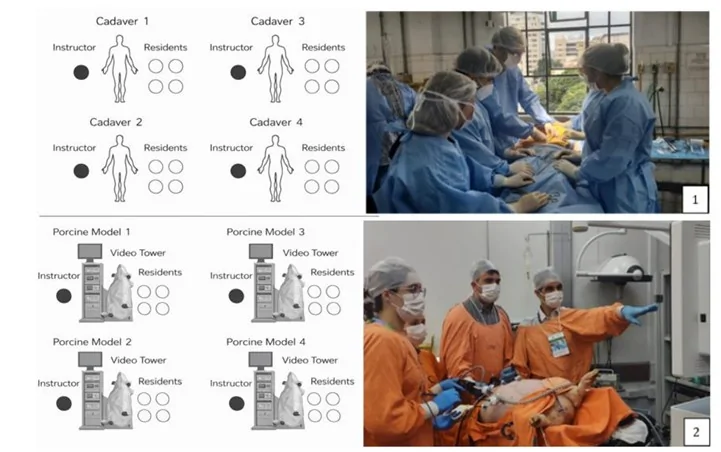
Practical section in the Medicine School of the University of São Paulo. The upper panel illustrates the cadaveric training stations, each supervised by one instructor and attended by small groups of residents. The lower panel shows the porcine model stations for laparoscopic activities, also supervised by an instructor. This structure allowed simultaneous hands-on training with close supervision during both practical stations of the course (pictures 1 and 2).

Source: personal archive

 Medtronic Brasil^®^: In this module, residents received three hours of instruction on surgical device handling followed by four hours of black-box practice. Exercises included object transfer, suturing, and manual anastomoses on synthetic materials via laparoscopy. 

### Resources and personnel

 Residents had access to open and laparoscopic instruments provided by FMUSP; orthopedic and special surgical materials were provided by Medtronic Brasil ^®^ (linear and circular staplers, cartridges, trocars, and ultrasonic devices). 

 The course involved 23 colorectal surgeons from FMUSP, one veterinarian, six technical assistants supporting animal and cadaveric models, three technicians supporting laparoscopic equipment, and four technicians supporting stapler handling. 

### Assessments

 Participants completed three questionnaires at different time points: one before the pre-course (Q1), another before the in-person course (Q2), and one at the end (Q3). 

 Q1: Included epidemiological data, a pattern of surgical exposure at the resident’s home institution, ten theoretical multiple-choice or short-answer questions on colorectal surgery, and self-confidence ratings (1–5) for the following procedures: 1–Open right colectomy (ORC), 2–Laparoscopic right colectomy (LRC), 3–Open left colectomy (OLC), 4–Laparoscopic left colectomy (LLC), 5–Total mesorectal excision (TME), 6–Mechanical ileotransverse anastomosis (MITA), 7–Mechanical colorectal anastomosis (MCA). 

 Q2 and Q3: Repeated the same ten theoretical questions to assess knowledge acquisition. Q3 repeated the confidence scale, collected overall satisfaction (0–10), and asked for written positive and negative feedback. 

 The responses were analyzed using R Commander^®^, employing non-parametric tests for independent samples (MannWhitney), due to the lack of identical respondent groups. 

## RESULTS

 Fifty-two first-year colorectal surgery residents participated. Questionnaire adherence rates were 77% for Q1 (40 responses), 86.5% for Q2 (45 responses), and 84% for Q3 (44 responses). Among the Q1 respondents, 21 (52%) were female, most were aged 28–31 years (65%), and half were from the Southeast region (Table 1). 

**Table 1:** Sample characteristics of Questionnaire 1 participants.

Variable/Category		n (%)^*^
Gender		
	Female	21 (52.5)
	Male	15 (37.5)
	Queer	1 (2.5)
Age range (years)		
	26–27	3 (7.5)
	28–31	26 (65.0)
	32–46	11 (27.5)
Region of origin (Brazil)		
	Southeast	20 (50.0)
	Northeast	10 (25.0)
	Central-west	4 (10.0)
	North	3 (7.5)
	South	3 (7.5)
Colorectal surgery residency location (region)		
	Southeast	26 (65.0)
	Northeast	8 (20.0)
	Central-west	5 (12.5)
	South	1 (2.5)
	North	0 (0.0)

* Values are expressed as number (percentage).

 Detailing prior experience, 85% reported exposure to minimally invasive surgery at their institutions, while 82.5% had never participated in robotic procedures (Table 2). The procedures most frequently performed monthly (≥10 cases) were anorectal surgeries (18 residents), open surgeries (ten residents), and, to a lesser extent, laparoscopic (eight residents) and robotic procedures (one resident). Exposure to minimally invasive surgery was greatest among residents from the Southeast. 

**Table 2:** Distribution of responses regarding the number of monthly surgeries performed by colorectal surgery residents, according to surgical approach.

Number of surgeries/month	Transanal n (%)	Open n (%)	Laparoscopic n (%)	Robotic n (%)
≥10	18 (45.0)	10 (25.0)	8 (20.0)	1 (2.5)
5–10	16 (40.0)	20 (50.0)	9 (22.5)	1 (2.5)
1–5	6 (15.0)	10 (25.0)	17 (42.5)	5 (12.5)
0	0 (0.0)	0 (0.0)	6 (15.0)	33 (82.5)

 Regarding self-confidence, residents reported greater confidence in open (“conventional”) procedures compared with minimally invasive (“laparoscopic”) ones, both at the beginning and the end of the course (p<0.05). A significant improvement was observed after training, especially for laparoscopic right colectomy (p=0.0043), laparoscopic left colectomy (p=0.00048), and total mesorectal excision (p=0.00023) (Table 3 ). 

**Table 3:** Residents’ confidence level for performing colorectal procedures before and after the course (1–5 scale).

Procedure	Mean±SD (pre)^*^	Mean±SD (post)^*^	p-value^†^
Open right colectomy	3.65±0.98	3.85±0.92	0.38478
Laparoscopic right colectomy	1.65±0.98	2.20±0.94	0.00430
Open left colectomy	3.58±1.08	3.70±0.97	0.60029
Laparoscopic left colectomy	1.52±0.93	2.15±0.86	0.00048
Total mesorectal excision	1.70±1.04	2.50±0.96	0.00023
Mechanical ileotransverse anastomosis	3.58±1.13	3.90±1.01	0.20090
Mechanical colorectal anastomosis	3.27±1.15	3.67±1.05	0.14224

* Values are expressed as mean±standard deviation (SD)

† Statistical analysis: Mann-Whitney test.

Confidence was assessed using a 5-point Likert scale.

 For theoretical knowledge, mean scores increased progressively: Q1=6.74; Q2=7.53; Q3=7.95. The improvement was statistically significant after the pre-course (Table 4). 

**Table 4:** Participants’ performance in the theoretical assessment at the end of each course stage.

Comparison	Mean score Q1^*^	Mean score Q2^*^	Mean score Q3^*^	p-value^†^
Q1 vs. Q2	6.74	7.53	—	0.0113
Q1 vs. Q3	6.74	—	7.95	0.00023
Q2 vs. Q3	—	7.53	7.95	0.233

* Values are expressed as mean scores;

† Statistical analysis: Mann-Whitney test.

Q1=Questionnaire 1 (baseline assessment); Q2=Questionnaire 2 (after pre-course theoretical phase); Q3=Questionnaire 3 (after the practical course).

 Mean satisfaction score was 9.3. The most frequently cited positive aspects included:

1. Opportunity for supervised practical training with specialists,
2. Technical and didactic quality of instructors,
3. Prioritization of practical activities with earlier theoretical review.

Among the limitations, the residents highlighted the need for more instructors and additional practical time (Table 5). 

**Table 5:** Positive and negative aspects of the course from the residents’ perspective.

Positive aspects		Number of responses
Positive aspects		
	Opportunity for hands-on training supervised by specialists	18
	Technical and teaching quality of the instructors	17
	Emphasis on practical activities with theoretical content reviewed before the course	16
	Quality of simulation models (porcine and cadaveric)	11
	Relevance and applicability of the topics covered	10
	Course organization and structure	9
	Access to materials not routinely available in daily practice	6
	Interaction with professionals from other institutions	3
Negative aspects		
	Insufficient time for hands-on practice	12
	Theoretical activity at medtronic ^®^ reduced practical training time	9
	Lack of an exclusive instructor for each group in the porcine model	7
	Theoretical content was offered only once	4
	Unequal training time among participants	1
	Absence of laparoscopic training using cadaveric models	1
	Dry lab training didn’t precede wet lab training for all residents	1
	Low table height during training	1
	No video-based practice of right and left colectomy steps	1

## DISCUSSION

 The hybrid training model used in this study resulted in gains in knowledge and confidence among the residents in the specialty. Traditionally, during surgical training, a resident’s first contact with operative technique often occurs directly in a patient^18^ . In this setting, the intraoperative environment imposes high stress, requiring experienced surgeons to ensure procedural quality and safety regardless of complexity. Cosequently, the learning of basic surgical techniques may be compromised^2^ . Simulation centers therefore emerge as fundamental tools^10 ,18^. Until now, no literature has described a structured, free colorectal surgery simulation program similar to the one proposed in this study. 

 Surgical training models may be organic (animal or cadaver) or inorganic (synthetic or computer-based, including virtual reality)^21^ . Limited availability and cultural or religious barriers may restrict the use of cadaveric models^22^ . However, despite advances in high-fidelity synthetic or electronic 3D models, none have surpassed cadaveric models in providing a high-quality surgical experience^7,11,15,17^. In this course, students had access to varied training models, especially fresh cadavers, which offer maximal human realism and superior quality compared with formalin-preserved specimens^21^ . Although cadavers facilitate anatomical study, they poorly simulate bleeding and hemostasis. This limitation was addressed through the use of live animal models^21^ . Participants underscored the value of organic models as a strong positive aspect of the course. 

 There is increasing integration of technology into therapeutic procedures^2^ . Laparoscopy offers advantages over conventional open access, including less postoperative pain, shorter hospital stay, and earlier return to daily activities^24,25^, with equivalent oncologic safety^14,24^. In this study, although most participants (85%) were exposed to laparoscopy during residency, nearly half (42.5%) reported low frequency (one to five procedures per month), and robotic platforms were rarely available. This limited exposure may prolong the learning curve, making the course particularly beneficial for such learners. 

 Most residents (65%) came from the Southeast region. This may reflect both a selection bias — since most registrants were also from this region — and historical inequalities that concentrate advanced surgery in university or private centers in southern and southeastern Brazil^4,8,16^. Even after four decades since the introduction of videolaparoscopy in Brazil, fewer than half of cholecystectomies performed in the Public Health System (SUS) were laparoscopic in 2022^1,19^. This limited access may explain residents’ greater confidence in open procedures and reinforces the need for broader investment in infrastructure and training, such as the initiative presented here. 

 Despite the growing importance of minimally invasive techniques, open surgical training remains essential^6^. An important finding of this study was the improvement in residents’ confidence in performing laparoscopic colectomies even without completing the entire procedure themselves. This resulted from the integration of theoretical instruction, open surgical steps, and laparoscopic exercises. Mastery of fundamental anatomical and technical principles remains the basis of surgical development^5^. When reviewing the literature, there is a lack of studies allowing standardized didactic teaching of advanced colorectal procedures^13^. 

 In 2013, Fonseca et al. highlighted the risk of training deficits in open surgery amid the rise of minimally invasive techniques. Although this does not reflect the reality of most Brazilian training centers, that study aligns with ours in emphasizing the value of conventional surgery training, which is scarcely described in the literature^9^. The inclusion of fresh cadaveric open procedures in this course aimed to meet this need by providing dedicated training time. 

 The high satisfaction rate, particularly regarding close supervision from specialists with strong teaching skills, deserves emphasis. The course provided a welcoming, safe, and differentiated environment in which the teacher-student relationship was highly valued, consistent with literature reporting that continuous and supportive expert feedback is essential for technical development and correction of deficiencies^21^. A multicenter international study in 2011, involving the Universidade Federal de São Paulo, showed frequent dissatisfaction among surgical residents with training content and operative volume, and only slightly more than half would seek their supervisors to address residency-related issues^12^. A recent study by Tee et al. evaluating the effectiveness of surgical simulation also concluded that instructor feedback is critical for achieving favorable outcomes^23^. 

 This study has methodological limitations — including the absence of paired groups and lack of long-term retention assessment — but the data suggest a positive immediate impact on training. It is important to emphasize that increased confidence after the course does not equate with procedural proficiency. Single exposure to training models such as those described here does not guarantee long-term results, which are essential for professional development. 

 Nevertheless, initiatives like this, supported by well-regarded medical schools and experienced professionals, enable residents with heterogeneous academic and clinical backgrounds to achieve integrated and inclusive learning gains while inspiring new training centers. Future studies should include longterm follow-up and periodic repetition of the course to assess the consolidation of learning. 

## CONCLUSIONS

 The Advanced Practical Course in Colorectal Surgery proved to be an innovative and impactful initiative in the training of colorectal surgery residents in Brazil. The use of realistic models, combined with a hybrid teaching methodology, enabled the development of technical skills in a safe environment. Supervised and supported by solid institutions and academic disciplines with aligned educational and social goals, the course demonstrated positive applicability and measurable knowledge gain. 

 The significant improvement in participants’ confidence in complex procedures, along with their high satisfaction, confirms the model’s effectiveness. The expansion and continuity of similar initiatives are essential to increase access to structured training and strengthen new centers of excellence in surgical education across the country. 

## Data Availability

The datasets generated and/or analyzed during the current study are available from the corresponding author upon reasonable request.
